# Combining models to generate a consensus effective reproduction number *R* for the COVID-19 epidemic status in England

**DOI:** 10.1017/S0950268824000347

**Published:** 2024-03-14

**Authors:** Harrison Manley, Josie Park, Luke Bevan, Alberto Sanchez-Marroquin, Gabriel Danelian, Thomas Bayley, Veronica Bowman, Thomas Maishman, Thomas Finnie, André Charlett, Nicholas A Watkins, Johanna Hutchinson, Graham Medley, Steven Riley, Nowcasts Model Contributing Group, Jasmina Panovska-Griffiths

**Affiliations:** 1 UK Health Security Agency, London, UK; 2 University College London, London, UK; 3 Defence Science and Technology Laboratory, Fareham, UK; 4 The Nowcasts model contribution group comprises Sebastian Funk (LSHTM, London, UK), Paul J Birrell and Daniela De Angelis (UK Health Security Agency and MRC Biostatistics Unit, University of Cambridge, Cambridge, UK), Matt Keeling (University of Warwick, Coventry, UK), Lorenzo Pellis (University of Manchester, Manchester, UK), Marc Baguelin (Imperial College London, London, UK), Graeme J Ackland (University of Edinburgh, Edinburgh, UK), Jonathan Read and Christopher Jewell (University of Lancaster, Lancaster, UK), and Robert Challen (University of Exeter, Exeter, UK); 5 The Big Data Institute and the Pandemic Sciences Institute, University of Oxford, Oxford, UK; 6 The Queen’s College, University of Oxford, Oxford, UK; 7 London School of Hygiene and Tropical Medicine, London, UK

**Keywords:** COVID-19, reproduction number R, ensemble modelling, statistical analysis

## Abstract

The effective reproduction number 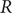 was widely accepted as a key indicator during the early stages of the COVID-19 pandemic. In the UK, the 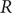 value published on the UK Government Dashboard has been generated as a combined value from an ensemble of epidemiological models via a collaborative initiative between academia and government. In this paper, we outline this collaborative modelling approach and illustrate how, by using an established combination method, a combined 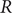 estimate can be generated from an ensemble of epidemiological models. We analyse the 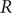 values calculated for the period between April 2021 and December 2021, to show that this 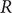 is robust to different model weighting methods and ensemble sizes and that using heterogeneous data sources for validation increases its robustness and reduces the biases and limitations associated with a single source of data. We discuss how 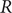 can be generated from different data sources and show that it is a good summary indicator of the current dynamics in an epidemic.

## Introduction

Since the onset of the coronavirus disease in early 2020 (COVID-19) as a pandemic, mathematical modelling has been widely used to generate policy-relevant evidence. Mathematical modelling provides a framework for simulating the dynamics of the pandemic. When parameterized with and calibrated to data, this can be used to generate projections of future epidemic trajectories as well as to track the current epidemic status. Epidemiological estimates such as the reproduction number 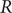 derived from models can be useful tools for such epidemic status tracking.

The reproduction number 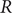 is a measure of the infectious potential of a disease and represents the average number of secondary infections that emerge from one infection [1]. At the onset of a new disease, in a naive, fully susceptible population, the basic reproduction number 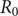 represents the average number of secondary infections stemming from an initial case. In contrast to 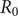 , 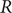 is the reproduction number at any time during an epidemic – often referred to as the *effective reproduction number* 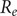 or *temporal reproduction number* 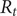 [2]. It reflects the average number of secondary infections generated from a population consisting of susceptible, exposed, and immune individuals, and potential changes in mixing and the presence of interventions.

The growth rate 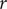 represents the rate at which the epidemic is growing during the exponential phase of epidemic growth. In epidemiological modelling, 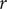 and 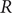 are related via the generation time distribution 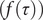 of the epidemic [2]. Mathematically, this is expressed as follows:
(1)

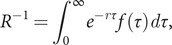

where 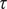 is the time since infection of an individual and the generation time distribution 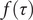 is defined as the probability density function for the time of a subsequent infection made by that individual. The generation time T_g_ is defined as the mean of the generation time distribution. As evident from this equation, changes in 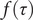 , 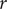 _,_ and 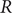 affect each other. Although precise statements depend on the specific shape of 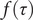 , broadly speaking, for fixed T_g_, r and R increase/decrease in tandem; for fixed r, T_g_ and R increase/decrease in tandem, while for fixed R, increasing T_g_ means decreasing r and vice versa.

While 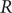 is reflective of the current strength of transmission, 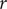 is reflective of the transmission speed [3]. Both provide information about the impact of control measures. For example, if an intervention is imposed and 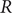 is consequentially reduced to below the 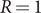 threshold, or 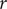 is reduced to below the 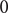 threshold, this suggests that the intervention has had an impact on reducing onward transmission. However, when providing policy advice during the COVID-19 epidemic, 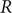 was used as it is more easily interpretable than 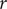 and does not require a conceptual understanding of exponential growth or decay, so it is therefore simpler to explain to the public. Additionally, 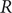 at the onset of the epidemic 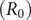 provides information on the likely level of herd immunity necessary. In a homogeneous population, the herd immunity threshold as a percentage of the population, 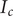 , can be calculated as follows:

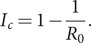

which suggests that the more people that become infected by each individual who has the virus, the higher the proportion of the population that needs to be immune to reach herd immunity [4]. However, it should be noted that this is subject to large uncertainties due to the difficulty in calculating 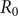 , which leads to differing estimates of 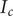 , and should therefore be used with care [5, 6]. Further details on 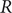 and the differing methodologies for calculating the reproduction number can be found in the Section titled “Outline of epidemiological models used to produce 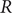 values”.

In the UK, the Scientific Advisory Group for Emergencies (SAGE) is activated in response to emergencies and is made up of several subgroups consisting of experts relating to different scientific fields [7]. These subgroups are often called upon in order to provide evidence to the UK government relating to key policy questions. One of these groups is the Scientific Pandemic Influenza Group on Modelling-Operational (SPI-M-O), which has been leading the modelling of the COVID-19 epidemic since its onset [8]. SPI-M-O primarily consists of experts in infectious disease modelling.

In early 2021, a formal collaboration between SPI-M-O and the UK Health Security Agency Epidemiological Ensemble (UKHSA Epi-Ensemble) modelling group was established, which has provided the UK government with weekly estimates of key epidemiological indicators, including the effective reproduction number 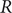 [9] throughout 2021–2023. The consensus values were generated as a combined estimate from a set of epidemiological models maintained and run by members of SPI-M-O and the UKHSA Epi-Ensemble and were combined using a random-effects meta-analysis approach with equal weighting applied [10], with visualization implemented using CrystalCast developed by the Defence Science and Technology Laboratory (DSTL) [11]. The combined estimates were agreed in a weekly meeting of the UKHSA Epidemiology Modelling Review Group (EMRG), attended by government modellers and policy stakeholders, as well as academic modellers.

Generating a combined ensemble estimate in place of a single model truth can lead to improved predictive power [12], allows an increased robustness of the outcomes, and is a useful tool for policymakers [13]. Generating a combined estimate from a set of models is not a new concept; they are widely used across many disciplines, such as forecasting the weather [14], hydrology [15], flood losses [16], cancer prediction [17] and climate modelling [18]. Within infectious diseases, combined model estimates have been applied to modelling human immunodeficiency virus (HIV) [19], influenza [20], and Ebola [21, 22] transmission and recently for outbreak analysis related to COVID-19 in the United States [23] and Europe [24].

While mathematical models have been used to offer informed advice to the scientific community and policymakers throughout the COVID-19 pandemic across a number of countries, the use of modelling has differed. For example, modellers in the United States, in conjunction with the Center for Disease Control and Prevention (CDC), published ensemble forecasts using a wide variety of mathematical models [13, 25]. These models had focused on forecasting new cases, hospitalizations, and deaths at a national and state level but did not estimate 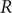 or 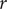 specifically. On the other hand, in New Zealand and Italy, modellers advising the government have compared estimates of 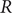 obtained from different models but without producing formal combined estimates [26, 27]. In Norway, multiple data sources including confirmed cases, proportion of COVID-19 attributable hospital admissions, and a national symptom survey were used to estimate 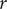 over the course of the pandemic, but only one model has been used to estimate 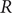 from these sources [28]. Similarly, the Robert Koch Institute in Germany only used a single model to estimate 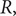 which depended on nowcasting estimates of the number of new cases [29].

As noted above, in the UK, since the onset of the pandemic, a set of mathematical models developed, maintained, and applied by the members of SPI-M-O and the UKHSA Epi-Ensemble have been used to track the epidemic status, including generating 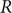 and 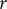 alongside estimates of incidence and prevalence. The 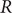 value published on the UK Government Dashboard [30] has been generated as a combined value from these models and agreed at the weekly EMRG meeting.

The usefulness in getting a combined estimate from across models and data sets is not just in the averaging of different models’ estimates with weighting but also in the formation of a community that is constantly discussing the outcomes, the assumptions, and the input data identifying the drivers behind the differences across models. This is especially important when generating 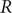 . While doubling time and 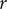 can be thought of as almost features of the data, requiring very few assumptions, the move to 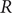 requires a set of subjective assumptions. This is why there is a need to have multiple groups making different assumptions, leading to heterogeneous outcomes that can be discussed, understood, and combined. When 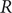 can be generated using different data sets, in addition to different models, this is particularly important. The development of the formal collaboration between the modellers at UKHSA Epi-Ensemble and within SPI-M-O, and the weekly technical meetings of the group and the follow-up EMRG meeting, gave a platform for informed discussions of the similarities and the differences across models’ nowcast estimates and provided a place where decisions could be made on whether to include or exclude a given model from the combined estimate.

This paper outlines the process of this collaboration between government and academia to continually generate estimates for the effective reproduction number in England over the COVID-19 epidemic. Specifically, we outline how a previously established combination method, described in [10], has been applied in the UK throughout the COVID-19 pandemic. We detail our approach of generating a consensus value of 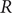 from an ensemble of epidemiological models applied to the English epidemic. We illustrate the process, show how a combined 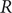 estimate has been generated in April 2021 and in September 2021, and explore the robustness of the combined 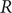 value on the size and weighting of the models’ combination. By comparing the change in 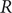 with the change in measurable data such as COVID-19 cases, hospitalizations, and deaths, we also explore whether 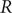 can be a good indicator of epidemic status.

## Methodology

### Outline of epidemiological models used to produce 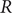 values

Generating an 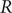 estimate requires a model of some kind with subjective assumptions and information from other sources. Our modelling ensemble comprised mathematical models that were developed, adapted, and used throughout 2020–2022, to model the COVID-19 epidemic in England; to generate epidemic metrics such as 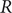 , 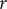 , incidence, and prevalence; and to produce medium-term projections (MTPs) of hospital admissions, hospital bed occupancy, and deaths. The MTPs will be explored separately in a future publication. These models fall into three broad groups, as described in [31] and [2]: population-based models (PBMs), data-driven models (DDMs), and agent-based models (ABMs). The models in the ensemble can be split further into three broad categories based on the data they primarily used to inform their estimates: case-based models, admission-based models, and models that were fitted to both case data and hospital data. For the purposes of this study, models that were fitted to survey data are categorized as case-based models as they were focused on detecting the incidence of the disease, though there were differing delays associated with models that were fitted to cases and models that were fitted to survey data. There are drawbacks and advantages associated with fitting to either cases or admissions. Case data are highly sensitive to ascertainment biases. For example, an under-ascertainment of cases may be related to weekend/weekday periods, with people with milder symptoms over the weekend less likely to get confirmed than during the weekdays. The scale of these biases has varied greatly over time. Therefore, models that were fitted to case counts or positivity must be interpreted in the context of testing behaviours and policies at the time. However, admission data are not free from bias either, as they depend on input from physicians and other hospital staff, which means that weekend/weekday effects are likely. In addition, the likelihood of being admitted to the hospital varies greatly by age. Hence, without age stratification in the model, it is likely that community transmission is underestimated among younger age groups. Furthermore, the delay between being infected with COVID-19 and being admitted to the hospital is on average far greater than that between infection and receiving a positive test. This presented difficulties when trying to produce timely estimates of community transmission. Table 1 lists these models along with the type of data they were fitted to and whether or not they were run internally by either the UKHSA Epi-Ensemble or a Devolved Administration (DA) Department.

**Table 1.** The UKHSA/SPI-M-O models split by model type and the data to which they fit to

Model name	Model type	Data type	Was the model run either by the UKHSA or a DA department?
Lancaster Spatial Stochastic	PBM	Case data	No
Edinburgh WSS Model	PBM	Case data	No
Manchester Model	PBM	Hospital data	Yes
University of Liverpool Model	PBM	Hospital data	Yes
PHE/Cambridge	PBM	A mix of data	Yes
Warwick Model	PBM	A mix of data	No
Imperial Model	PBM	A mix of data	No
EpiEstim	DDM	Case data	Yes
GenomicSurveillance	DDM	Case data	Yes
Epidemia	DDM	UKHSA runs two versions, one fitting to cases and one fitting to admission data	Yes
LSHTM EpiNow2	DDM	Two versions are run: one fitting to cases and one fitting to admissions	Yes
LSHTM ONS inc2prev	DDM	Fits to ONS positivity, which is treated as case data	Yes
Oxford CSML Model	DDM	Case data	Yes
OpenABM	ABM	Hospital data	Yes
Covasim	ABM	The UKHSA version fits to a mix of data	Yes

While these models can be broadly stratified into the PBM, DDM, and ABM groups, each model within the group has distinctive characteristics. For example, EpiEstim followed the methodology described in [32] and therefore assumed a consistent relationship between infections and cases. The estimated 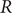 was therefore only robust when the ascertainment rate was roughly constant. While GenSur shared this same limitation, Epidemia and OxfordCSML did not make this assumption [33]. Furthermore, renewal equation-based models tend to be semi-mechanistic, that is assuming that the effects of interventions are absorbed into the data to which they fit. In contrast, fully mechanistic models, such as the susceptible–exposed–infected–recovered (SEIR) population-based models and ABMs, explicitly modelled the effects of interventions such as test–trace–isolate strategies and imposing and removing of social distancing measures.

In epidemiological models, the structure of the model determines the method to calculate 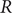 and depends on the assumptions and data sets used to parameterize and validate the model [34].

In the classic compartmental SEIR model, 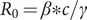 _,_ where 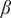 is the transmission probability per contact, 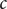 is the number of contacts 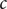 _,_ and 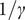 is the infectiousness period (average time that an individual is infectious for). 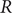 is typically calculated from more complex (e.g. multi-group) SEIR models as the largest eigenvalue of the next-generation matrix (NGM), which can be expressed as 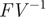 , where 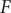 represents infection rates and 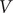 represents recovery rates [35, 36]. 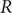 can also be estimated using the renewal equation [37, 38]:
(2)

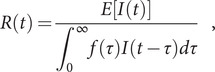

where 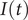 is number of new infections (i.e. the incidence) at time 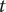 and 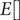 denotes the expected value.

Across the dynamical models that comprise sets of differential equations in our ensemble, such as the Manchester or University of Liverpool models, 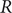 was estimated by inferring the rate of transmission within the model, which was fitted to observed data on cases, hospitalizations, deaths, or their combination. Some more complex dynamical models, such as the one developed by Public Health England (PHE) and Cambridge or the Imperial one (sircovid), explicitly calculated R as the largest eigenvalue of the NGM.

There is also a difference in how R was estimated between compartmental and ABMs or individual-based models. In ABMs, such as Covasim, it is possible simply to count exactly how many secondary infections are caused by each primary infection at any stage of the epidemic and hence explicitly calculate 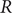 .

A third approach, and a characteristic of the data-driven models in our ensemble, used statistical models to estimate R empirically from the notification data. These methods made minimal structural assumptions about epidemic dynamics and only required users to specify the generation time distribution. A selection of models in this category in the ensemble was formulated based on Equation (1). For example, where the generation time distribution is described by a gamma distribution with shape 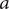 and rate 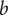 , 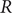 can be expressed in terms of the growth rate 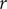 as follows:
(3)

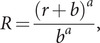



A high-level description of the methods used to calculate 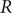 , along with an outline of the main characteristics of each model, is given in Table A1 in the Appendix.

### Combining model estimates to generate a consensus 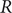


To generate combined 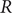 estimates from the ensemble of models, we used the statistical model developed as a collaboration between DSTL, the University of Southampton, and the University of Liverpool with the underlying methodology described in [10]. We present a high-level outline of the method below. Each of the epidemiological models described in Table A1 and calibrated to the data as outlined in Table 1 generated 5th, 25th, 50th, 75th, and 95th percentile estimates for 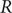 . Using these, a mean and a standard deviation for each model’s 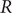 estimate were generated. The mean of the 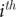 model, 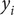 , was initially estimated as the median (or 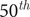 quantile), and the standard deviation was calculated as follows:
(4)

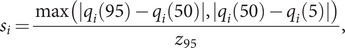

where 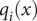 represented the 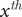 quantile of the 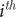 model and 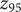 is the z-score for the 90% confidence interval (CI) of the standard normal distribution. Where model estimates were highly skewed, a skewness correction calculation was applied to provide alternative estimates for the mean and the standard error (see [10] for further details). Otherwise, the distribution of the model estimates for 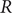 was assumed to be symmetric.

These estimates were then combined using a random-effects model, which allowed for differences in model structure and did not assume that models shared a common effect size. The random-effects statistical model was described as follows:
(5)

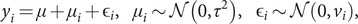

where the estimated mean for model 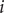 is denoted by 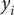 and the standard error is denoted by 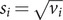 . The model was fitted to provide estimates for 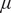 and 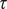 _,_ which are the mean and standard deviation of the true effect size, respectively. The between-model variance, 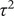 , was estimated using the restricted maximum-likelihood method, and the CI of the mean true overall effect size is estimated using the standard Wald-type method. The models were equally weighted (see next section for more details) and the range of 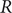 was rounded out to one decimal place, by using the lower and upper bounds, respectively. Further details of other methods used for calculating the between group errors and CIs are provided in [39].

### Collaborating across government and academia to produce a consensus nowcast

The process of cross-academic and government collaboration to generate consensus 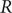 was done in several steps. Firstly, the outputs from the models detailed in Table A1 were submitted by the modellers to the UKHSA Epi-Ensemble team weekly. The team then combined the model estimates using CrystalCast to generate a combined estimate for 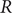 , 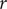 , incidence, and prevalence in England, the English regions and the DAs. The combined estimates, as well as individual model estimates, were discussed at a weekly meeting between the UKHSA Epi-Ensemble and SPI-M modellers, wider SPI-M-O members, and wider representatives from UKHSA and DAs. These meetings gave the modellers the chance to explain their outputs, discuss the model behaviour, and agree on the inclusion or exclusion of any specific models in the ensemble for that week. A model would only be excluded if there was a clear error in its outputs or if it displayed behaviour that could not be justified from an epidemiological perspective. Once a consensus was reached for each of the epidemic metrics, a recommendation was made to the EMRG, who then finally approved and published the consensus outputs.

### Sensitivity analysis

Two sensitivity analyses explored the extent to which the combined 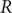 would have been impacted by the variable weighting of the models within the ensemble and the size of the ensemble. For consistency, no individual models were re-run for these analyses; we used only the original model results submitted at the time the consensus 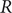 was published. This was intentional so that the analysis would serve as a historic record of the combined estimates at the time.

#### Exploring the impact of model weighting on the combined R

Firstly, we explored the impact of the choice of model weighting on the consensus 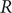 . The combined estimate 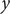 was calculated from the true effect size of each model 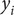 . The true effect size can therefore be weighted. The simplest method is equal weighting, which was used to generate the published consensus 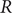 over 2020–2022. In this method, each model is assumed to have an equal contribution to the combined estimate under the assumption that all models are equally valid.

Another common method of weighting is inverse-variance weighting. In this method, models with a high variance, that is those that are less certain, are penalized more than models with a low variance, that is more certain models. However, individual models have different methods of representing uncertainty, and a model that is more certain is not necessarily more likely to be accurate. Therefore, this method is not applicable here.

An alternative method of model weighting is to group models by either their structure or the data to which they fit. For example, models that may have a different structure but use the same data form a subgroup as described in Table 1. We explored the impact of this on the consensus 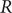 value by dividing the ensemble into subgroups, so each subgroup represents a homogeneous set of models according to either their structure or the data to which they fit. Models within each subgroup were equally weighted, and then, the contributions from the subgroups were equally weighted to give the overall combined estimate. This had two purposes: firstly, a single data stream or model structure would not have gained a larger weighting in the final combination, meaning that the combination was ‘data-agnostic’ or ‘model-agnostic’ and models such as EpiEstim, with a larger representation in the ensemble, did not bias the final estimates; secondly, it allowed us to compare the difference in trends between admissions and case data and therefore learn about the epidemic dynamics by inspection.

Similarly, as for the equal weighting model method, a consensus 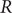 value was derived with this alternative variable weighting method as a range for April and September 2021. We present the results as rounded to two decimal places. However, we note that the range was published to only one decimal place to avoid presenting a false sense of precision. The range published was also rounded out, rather than rounding to the nearest decimal place, in order to increase the uncertainty instead of possibly reducing it.

#### Exploring the impact of ensemble size on the combined R

The models included within the ensemble varied throughout the pandemic; as new models were developed and introduced, some were phased out and others were updated in response to the changing epidemic. This could hypothetically result in inconsistent estimates through time. Furthermore, as UKHSA moved from a ‘response’ to a ‘business-as-usual’ phase during 2022, a need emerged to reduce the resource dedicated to modelling COVID-19 and hence reduce the number of models in the ensemble. These factors motivated us to explore how the combined 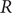 may have changed if a different model ensemble was used to generate it.

We investigated the implications of reducing the size of the ensemble on the combined 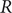 estimate over the period April 2021–December 2021. UKHSA models are labelled in Table 1 and comprised of internal models, that is run by UKHSA or DA modellers. We re-calculated the combined estimate using the ‘reduced’ ensemble of only internal models and, using equal weighting, compared this to the published consensus 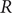 number in England.

### 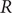 as an epidemic indicator

The 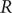 time series is a transform of epidemic metrics such as case incidence or hospitalizations. Hence, we expect it would be statistically correlated with the epidemic metrics, but quantifying the degree of correlation with different metrics is interesting.

We explored the correlation between the consensus 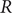 as published on the UK Government COVID-19 Dashboard and the key public data sources relating to the COVID-19 pandemic, namely cases, admissions, and deaths. We expect the 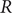 number to be correlated in some way to the rate of change of these three metrics, and we know this relationship is non-linear. Therefore, we used Spearman’s rank correlation coefficient, 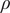 .

In order to adjust for periodic weekly fluctuations (e.g. weekend/weekday differences in under-ascertainment), each source of data was transformed into a centred weekly moving average. For each date that an 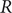 number was calculated, the slope of the data was calculated over a centred weekly window. We used the same length and position of windows over which to perform the analysis in order to ensure consistency; otherwise, additional artificial lag would be introduced into the analysis.

The correlation between 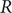 and the weekly rate of change in cases, admissions, or deaths may have an inherent lag due to the fact that it takes time for more severe symptoms to develop. In order to investigate this, we explored how the correlation changed between the 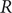 number, shifted along its time axis by a varying number of days, and the rate of change of new hospital admissions and deaths. This was done by shifting the calculated values of 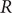 we used by 1–20 days and observing how 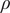 changed with an increasing shift size. Mathematically, we are calculating the following, where the variable 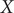 represents the centred weekly rolling average of either the recorded incidence of cases, hospital admissions, or deaths:

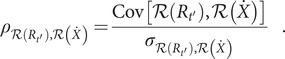



In the above, 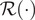 denotes the ordinal rank and 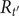 is the time-shifted 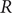 , equal to 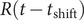 _,_ where 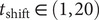 . 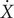 denotes the rate of change of variable 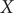 with respect to time, and all times considered are measured in days. We performed this calculation on data within specific time windows, which correspond to the Delta and Omicron waves, respectively, and shifted the time window for the published 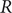 value against the static recorded data. These time windows were 7 May 2021 to 30 July 2021 and 26 November 2021 to 25 February 2022 for the Delta and Omicron waves, respectively.

## Results

### Generating a consensus 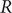 range in April and September 2021 using different weighting methods

**Figure 1.**
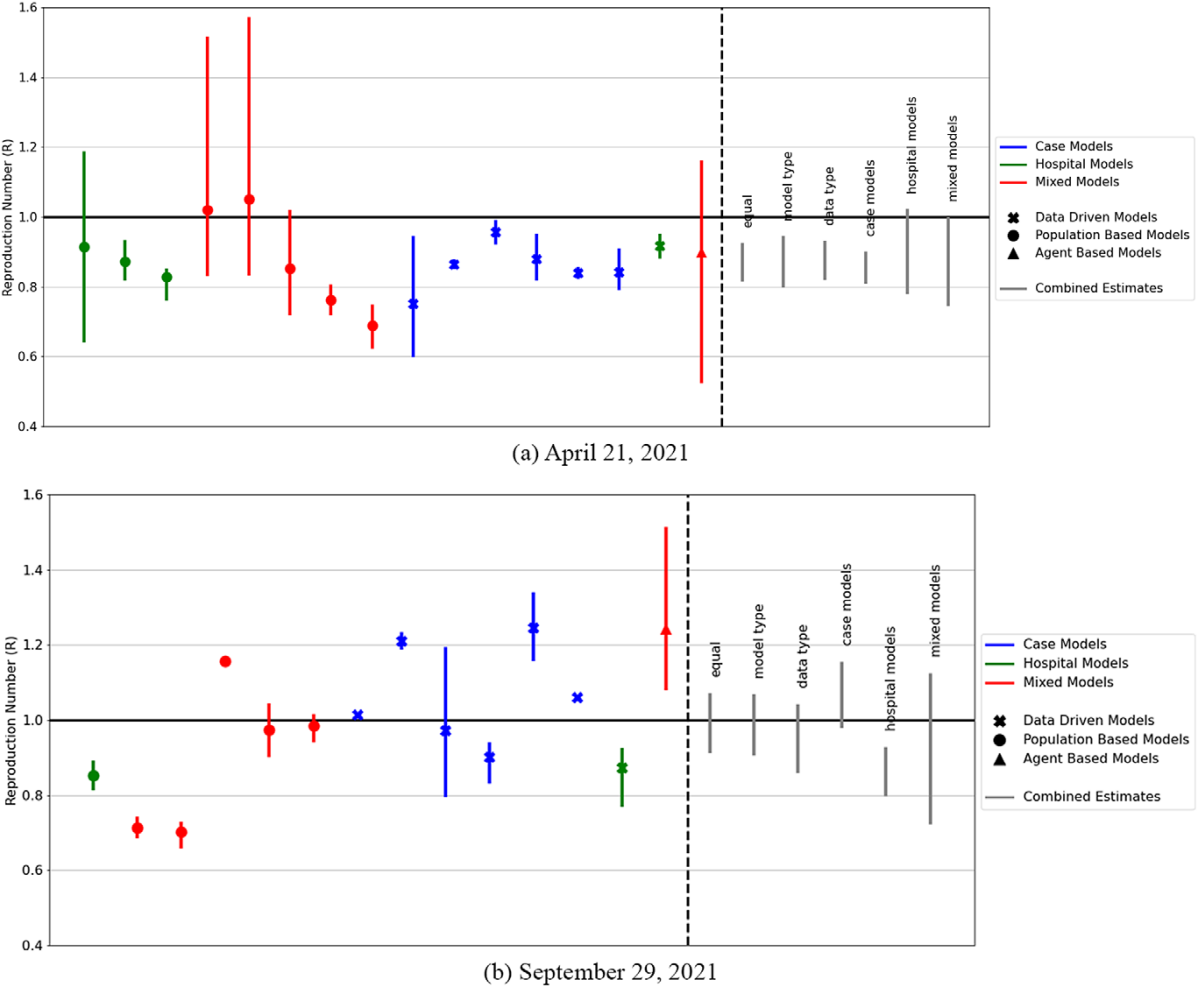
Model ensemble generated 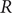 values at two time points of the COVID-19 epidemic in England. The parts of each plot to the left of the dashed line show the median and the 10th and 90th percentiles of the reproduction numbers 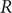 from the models included in the model ensemble on 21 April 2021 and 29 September 2021. The 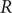 values on the right of the dashed line show the 90% CI for the combined 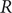 value generated with different weighted methods. Because of the delays between new infections and the time they are observed as cases or admissions, the combined R estimates reflect the R values on 6 April 2021 and 14 September 2021.

Whisker plots of the 90th CIs of 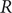 for each model are plotted alongside the resulting combinations from the different methods and shown in Figure 1. We note that because of the delays between new infections and the time they are observed as cases or admissions, the combined R estimates on 21 April 2021 and 29 September 2021 reflect the R values on 6 April 2021 and 14 September 2021.The numerical values for the 90% CIs for each weighting method are given in Table 2.

**Table 2.** 90% confidence intervals for combined 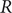 estimates using different weighting methods

Weighting methodology	6 April 2021	14 September 2021
Equal weighting	[0.81, 0.93]	[0.91, 1.07]
Weighting by data	[0.82, 0.93]	[0.86, 1.04]
Weighting by model	[0.80, 0.94]	[0.90, 1.07]
Equal weighting of case models	[0.81, 0.90]	[0.98, 1.15]
Equal weighting of hospital models	[0.78, 1.02]	[0.80, 0.93]
Equal weighting of mixed models	[0.74, 1.00]	[0.72, 1.12]

Using the equal weighting method, and combining the 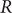 outcomes from the various epidemiological models (a mixture of SEIR-type, agent-based, and data-driven models), we generated combined 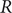 estimates of 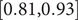 in April 2021 and 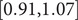 in September 2021. These represent the 90% CI that was published on the UK Government dashboard at the time.

Using a different weighting for the combination of models produces very similar combined 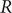 values at the two snapshots in time we studied: in April 2021 and in September 2021. Weighting by data resulted in an 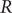 combination of 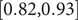 and 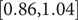 for the April 2021 and September 2021 estimates, respectively. Weighting by model structure resulted in a combination of 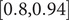 and 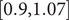 for the April 2021 and September 2021 estimates, respectively.

### The effect of ensemble size on the combined estimate

**Figure 2.**
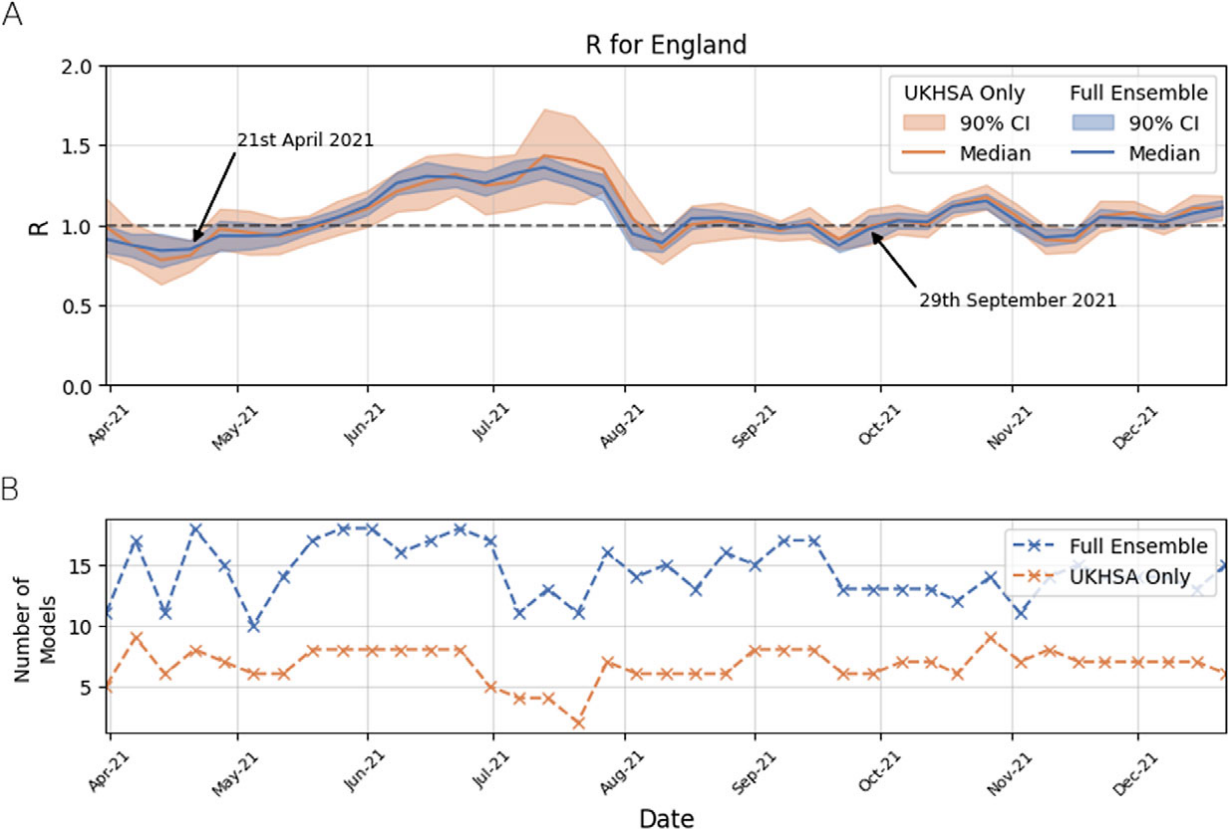
The combined 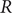 number in the period April 2021–December 2021 in England for the full model ensemble and the reduced (internal UKHSA and DA models only) ensemble. Plot A shows the time series of the two 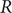 values over the study period, while plot B shows the number of models in each ensemble at different time points when the 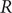 value was generated.

Figure 2A compares the unrounded combined 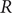 number generated from a reduced model ensemble that includes models run by UKHSA and DA teams as outlined in the sectiontitled “Exploring the impact of ensemble size on the combined R”. Our results show that the two combined 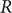 value time series are similar but not identical, with the level of agreement changing over the study period. For most of the study period, the values of the combined 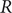 from the two model ensembles were similar, with the smaller model ensemble increasing the uncertainty in the consensus 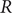 value (comparing the width of the blue bandwidth and orange bands in Figure 2A). There was a notable difference in the combined 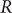 from the two ensembles in July 2021, which is due to a very different number of models constituting the model ensemble. The full ensemble for 14 July 2021 contained thirteen different models, compared to the internal model ensemble, which contained four different models (Figure 2B). On 21 July 2021, the full ensemble had eleven models, while the internal model ensemble contained only two. The two models for 21 July 2021 would not have been sufficient to produce a published combination,^1^ but we have shown the result here for completeness. From August 2021 onwards, the full and internal model-only ensembles show much better agreement, which is due to the latter having a more comparable number of models.

### The combined 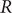 is a good, but delayed, epidemic indicator

**Figure 3.**
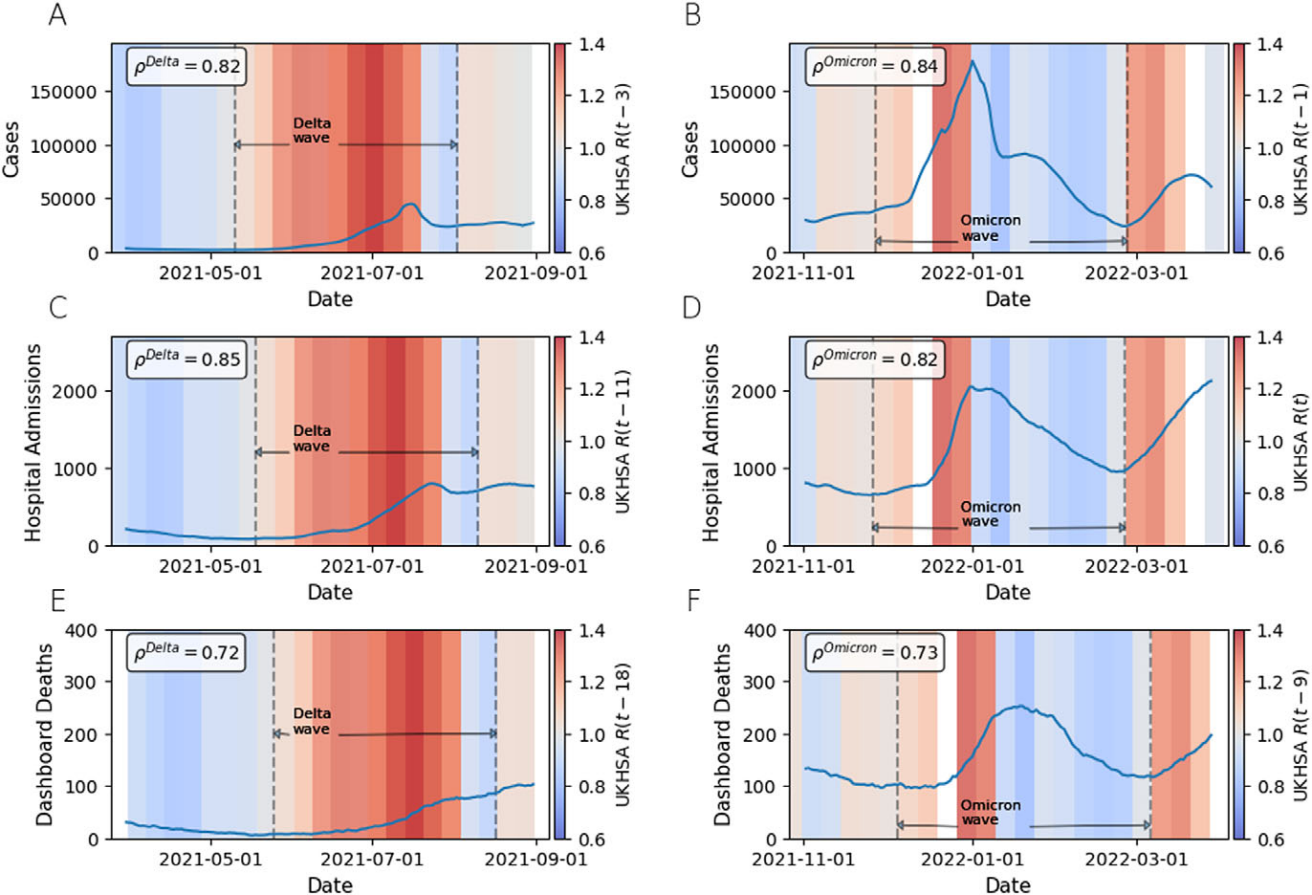
Plots comparing the published 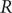 number to data published on the public government COVID-19 dashboard. The plots show the superimposed time series of the 7-day rolling average of the dashboard data for various metrics, on top of the published 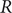 number for England. Where the shading is red, the median estimate for the 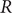 number was greater than 1. Where it is blue, the median 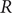 was less than 1. For each plot, Spearman’s rank correlation coefficient, 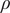 , was calculated to evaluate the correlation between the rate of change of the rolling 7-day mean of a given epidemic metric (cases, hospital admissions, and deaths) and the median published 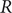 number, where 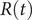 has been shifted along the time axis to maximize the correlation and 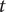 is measured in days. The amount of shift is different for each metric and wave. The maximum 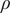 is obtained at a shift of 3 days for the Delta wave and 1 day for the Omicron wave for cases; 9 days for the Delta wave and no shift for the Omicron wave for hospital admissions; and 18 days for the Delta wave and 9 days for the Omicron wave for deaths. Only the data within the dotted lines pertaining to the Delta and Omicron waves, respectively, were included in the correlation calculation.

**Figure B1.**
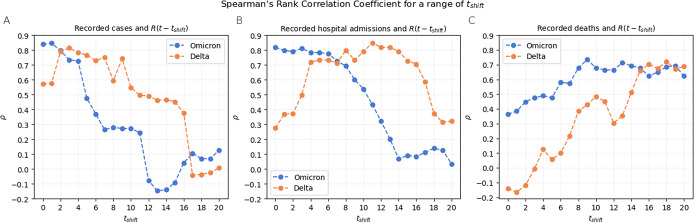
Plots A, B, and C show Spearman’s rank correlation coefficient, 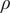 , between 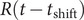 and the rate of change in cases, hospital admissions, and deaths, respectively, for a varying 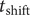 . The maximum value of 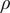 found from this analysis is included in Figure 3. The minimum *p*-values occurred in each instance for the maximum correlations; hence, the p-values are not included in this plot.

Figure 3 shows the relationship between the rate of change of the 7-day rolling mean of cases, admissions, and deaths with an optimally time-shifted 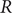 . Figure 3A shows that 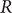 values larger or smaller than 1 (shown in red and blue, respectively) occur when the number of COVID-19 cases is increasing and decreasing. The correlation, calculated as Spearman’s rank coefficient between a time-shifted 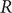 and the rate of change of recorded cases (Table 3), is given in the box to the top left of the plot. This is done separately for the Delta and Omicron waves, and the time periods we considered for each wave are demarcated by the vertical dotted lines. Overall, our results show a good positive correlation between epidemic status indicators and a time-shifted 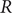 across both epidemic waves, confirming that 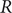 is following the trends in cases, hospitalizations, and deaths related to COVID-19 over both of the Delta and Omicron epidemic waves, albeit with a delay. Here, we have shown only the maximum correlation obtained from the optimal shift of the 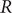 number. The values of 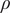 calculated for 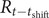 where 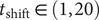 are shown in Figure B1.

**Table 3.** Spearman’s rank coefficient, 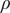 , and the respective *p*-values between the time-shifted 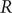 and the rate of change in a given epidemic metric. The coefficient was calculated only on data within the time period shown in the table

Metric	Dominant variant	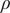	*p*-value	Days shifted
Cases	Delta	0.82	0.00003	3
Cases	Omicron	0.84	0.00064	1
Hosp. admissions	Delta	0.80	0.00002	8
Hosp. admissions	Omicron	0.83	0.00114	0
Deaths	Delta	0.72	0.00111	18
Deaths	Omicron	0.71	0.00654	9

## Discussion

This study outlines a collaborative approach across government and academia to generate the combined 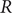 value for England over the period April 2021 to December 2021 using a previously established combination method [10] and 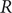 estimates from an ensemble of epidemiological models. The combined 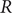 value was used to track the epidemic status over the COVID-19 epidemic in England and was produced by SPI-M-O in 2020 and by the UKHSA Epi-Ensemble modelling team since early 2021.

In this paper, we described the process of cross-academia and government collaboration and outlined the ensemble of epidemiological models used to generate individual 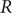 values in England, highlighting their key structural characteristics and the data they used, as well as the method to individually derive an 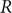 value. We also outlined the methodology developed in [10] of combining the individual 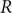 values to generate a combined consensus 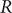 value and illustrated this by generating the published 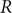 values of 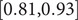 on 21 April 2021 and 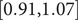 on 29 September 2021.

21 April 2021 and 29 September 2021 were very different epidemic points in time. 21 April 2021 followed the third national lockdown in England imposed to control the transmission of the Alpha variant [40]. Incidence and prevalence within the population were low and large-scale vaccination against COVID-19 had only started to be rolled out, with roughly half the population having received a first dose and only 8% having received a second dose. Against this mostly homogeneous immunity, susceptibility, and vaccine backdrop, the assumptions within the models would have been similar, producing similar 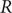 values across models.

In September 2021, the immunity, susceptibility, and vaccination levels were very different. There was a backdrop of population immunity from either vaccination or previous infection, with a large proportion of the population aged 12 and over either having received two doses of the vaccines or having been infected by the large Delta epidemic wave over the summer of 2021. The COVID-19 case rate remained high with schools just returning, and this period preceded the arrival of the Omicron variant.

Different models would have made different assumptions on the impact of the large Delta wave on population immunity and would have incorporated different assumptions around vaccination and social mixing associated with returning to school. All of these assumptions would impact individual 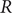 values, illustrated by the varying 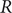 values across models at this time.

Furthermore, different models were fit to different data and this can generate different estimates. For example, the two London School of Hygiene and Tropical Medicine (LSHTM) EpiNow2 models, one that fits to cases and the second that fits to admissions, have vastly different 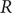 estimates. This difference is also reflected in the combinations from models that fit only to cases (reporting a range of [0.98, 1.15]) and from models that fit only to hospital data (reporting a range of [0.8, 0.93]). If we were only to use models that fit to cases, this would imply that the epidemic was increasing. However, models that fit to hospital data imply that the epidemic was decreasing. Models that fit to both report a central estimate in between the two with larger uncertainty. A more thorough study of different weighting methods and their effects on the combination estimate is out of the scope of this paper; however, this relatively simple example demonstrates that it is important that the ensemble features models that fit to a range of different data sources.

We note that the ensemble of models on 21 April 2021 and 29 September 2021 are not identical, and the model ensemble has been changing over time. New models were introduced to the ensemble throughout the epidemic, and models were omitted from or not submitted to the ensemble due to technical issues, such as calibration error or computer outage. Furthermore, in periods of change, such as the introduction of a new variant, some models required extensive development work before re-inclusion into the ensemble. This is an inevitable part of the process when collaborating with various modelling teams across government and academia, who are responsively modelling a fast-changing epidemic.

Reducing the size of the model ensemble to include only models run internally within UKHSA and DAs made a small difference to the combined 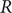 value, but did increase the width of the 90% CI. Overall, and for the majority of the study period, the values of the combined 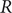 from the two model ensembles were similar as shown in Figure 2A. There were some differences around the peaks of the Delta epidemic waves in the summer of 2021, when the internal model ensemble (comprising UKHSA and DA-only models) had a very small number of models (Figure 2B) and as a consequence the combined 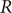 had a wider CI. This suggests that our process was robust to changes in the model ensemble, provided there were more than five constituent models going into any combination. This is encouraging for institutions that may be nowcasting future epidemic: an ensemble does not need to be enormous to reap the benefits of model combination.

The time series of the combined 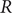 for the duration of the Delta and Omicron waves, respectively, is strongly positively and statistically significantly correlated with the rate of change of cases, hospitalization, and deaths related to COVID-19 (Figure 3). However, this strong positive correlation only occurred for each metric if the time series for 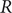 was shifted along its time axis by a certain optimum number of days, which differs for each wave and metric (Table 3). Exactly what causes the specific lag for each wave and metric is unclear. We acknowledge the limitations of using Spearman’s rank correlation coefficient to show this relationship; however, for this paper we simply wanted to gain an understanding of whether or not the 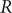 number is a valid proxy for epidemic status. Therefore, a more sophisticated regression model, combined with a full investigation of the cause of the lag between epidemic metrics and 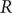 , is left to future work.

In order to mitigate uncertainty associated with nowcasting, since March 2021, the 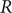 value from each model was taken on a single day in time 2 weeks before the day on which models were combined.^2^ Incorporating these delays in 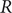 is important as not all models are always able to report estimates up to the day that they are run as they do not possess the ability to forecast. For example, the simplest model, an application of *EpiEstim*, uses a delay distribution between infection and the observation to which it is fit, to back-calculate and infer the incidence time series. The 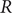 number is then estimated directly from the back-calculated time series for incidence. Therefore, the model is only able to provide estimates lagged to the order of the length of the delay distribution. Even where models are able to estimate current 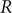 numbers, due to the delay between infection and observation, the infections occurring on a given day correspond to data that will be observed in the future and hence, are, in essence, projections. Due to the difficulty in producing accurate estimates for 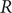 without the time delay, and in the light of the above discussion about the lagged correlation, it is vital to use a range of metrics to inform policy decisions around epidemic status. For this reason, the 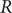 value estimates were used alongside estimates of three other epidemiological metrics when informing policy decisions: the growth rate, 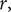 incidence, and prevalence, and the MTPs for hospital admissions, occupancy, and deaths.

### Future planning and lessons learnt

While combining multiple models, particularly in epidemic modelling, has proven to be very useful during the COVID-19 epidemic, there are lessons from this that should be considered in future.

Firstly, it should be ensured that CIs calculated by each of the models represent the same sources of uncertainty. Do they capture the underlying uncertainty present in the data, the parametric uncertainty or the structural uncertainty? The forecast hub at the CDC treats models primarily as black boxes, though model details are published and models are assessed for accuracy, and there is no explicit treatment of the resulting uncertainty. For future pandemics, there should be a clear definition of uncertainty and what it should represent.

Secondly, the combination method used to generate a consensus 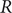 is insensitive to the performance of individual models. Whereas for forecasts, model performance can be calculated by comparing model estimates with observed data, the 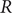 number is a latent variable and therefore is not observed. We rely on the expertise of modellers to ensure that models fit well to the data and make sound assumptions. In the future, developing an unbiased scoring method for individual models would help in ensuring the robustness and reliability of the individual models before combining them into an ensemble.

Finally, running an ensemble of models is resource-intensive and relies on a significant amount of external expertise. If models are not to be treated as black boxes, specialist expertise of academic groups continues to be required, and developing formal cross-government and academia modelling hubs is necessary for ongoing cross-institutional collaboration.

## Data Availability

The weekly published reproduction number 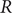 value data are publicly available at https://www.gov.uk/guidance/the-r-value-and-growth-rate. Individual model 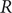 values can be requested from the corresponding author, but restrictions apply to the availability of these data, which were used under license for the current study, and so are not publicly available.

## Footnotes

1 A minimum of three distinct models were required for a combination to be published.

2 Prior to March 2021, SPI-M-O combined the most recent  numbers for each model, but found that there was little difference in the combined estimates produced by using estimates from two weeks prior [59].

## Code availability

The numerical code used in this analysis can be requested from the corresponding author. Restrictions apply to the availability of the model combining code, which was under collaborative license for the current study and hence is not publicly available.

## Appendix

### A models used in the ensemble

**Table A1.** Summary of the epidemiological models used to generate  outcomes for the English COVID-19 epidemic. We list the names of the models, their main modelling characteristics, and the data to which they are calibrated against and the method to calculate

Model name	Description	estimation
Lancaster Spatial Stochastic	A Bayesian spatial stochastic compartmental model that is fit to cases. Census commuter flow data are also used to infer mixing between local authorities	is calculated as the dominant eigenvalue of the NGM
Edinburgh WSS model [41]	The weight–shift–scale (WSS) model fits to case data to derive but accounts for systematic reporting errors (e.g. false positives and false negatives and under–reporting). Case counts are weighted, scaled, and shifted to account for the change in the size in future compartments, the delay between infection and case reporting, and to account for seasonality	The estimated is assumed to be the combination of the true plus a stochastic term and is calculated from the rate of change in reported cases, scaled by the time lag between infection and the time of case report
Manchester model (DetSEIRwithNB) [42, 43]	A deterministic compartmental ODE model that fits to hospital admissions, hospital occupancy, ICU occupancy, and deaths in hospital. , the transmission rate, varies step–wise between change points. Change points, such as policy or behavioural changes (e.g. schools returning and lockdowns), are defined by the modeller and are used to represent changes in the epidemic	is estimated from the most recent in the calibrated model
University of Liverpool model [44]	A Bayesian statistical model that comprises a deterministic compartmental transmission model governed by a system of ODEs and a stochastic observation model. Fitted to deaths, hospital admissions, and symptomatic report data from NHS 111 online	is calculated from estimates of the daily number of infections, the infectious population, and the mean time for which individuals are infectious
PHE/Cambridge model [45, 46]	A deterministic age–structured compartmental model fitted to serology data. Google mobility data are also used. Different versions of the model have been run throughout the pandemic that fit to slightly different data streams. Two versions of the model are presented in Figure 1. Deaths/ONS fits to ONS infection survey data, whereas regional/age does not. More recently, during the pandemic, the regional/age model has been replaced with the admissions/ONS model, which has the same model structure as deaths/ONS, but fits to admissions	is calculated as the dominant eigenvalue of the NGM
Warwick model [47]	A deterministic age–structured compartmental ODE model fitted to hospital and ICU admissions and COVID–19 positivity rate data	is calculated as the dominant eigenvalue of the NGM
Imperial Stochastic Compartmental (sircovid) [48]	Compartmental transmission model described by stochastic difference equations fitted to deaths, hospital admissions, and prevalence, tested cases in hospital beds, ICU prevalence, and serology data	is calculated as the dominant eigenvalue of the NGM
EpiEstim [32, 49, 50]	EpiEstim applies the renewal equation given a time series of incidence. The implementation described in [50] is used (code available at [49]). Estimates are back–calculated from an observation, for example cases, to time of infection, using an assumed delay distribution	is calculated using the renewal Equation (2)
GenomicSurveillance [51]	A spatio–temporal hierarchical Bayesian model, which fits to daily new cases and COVID–19 lineage counts	is calculated based on the derivative of the cubic spline function fitted to the incidence
Epidemia [52]	A hierarchical semi–mechanistic Bayesian model based on the renewal equation. Multiple data streams can be fit to simultaneously. Two separate versions of the model are run, one that fits to weekly admissions and one that fits to weekly cases. The admission version of the model was developed later in the epidemic in response to changes in the case ascertainment rate	is specified to vary weekly according to a random walk and is calculated using the renewal Equation (2)
LSHTM EpiNow2 [53, 54]	EpiNow2 uses the renewal equation to estimate , where initial infections are estimated based on the initial number of cases or hospital admissions. The relationship between cases (or hospital admissions) and infections is obtained from a convolution of the relevant delay distributions (an uncertain incubation period and reporting delay). Similarly to Epidemia, versions fitting to cases and admissions are run	is derived using the renewal Equation (2)
LSHTM ONS inc2prev [55]	A Gaussian process model that uses PCR positivity rates published by the ONS to model incidence by convolution with the curve estimating the evolution of the probability of a positive test since time of infection	is derived using the renewal Equation (2)
Oxford CSML Model Dashboard on [56]	Hierarchical semi–mechanistic Bayesian model fitted to cases, similar to Epidemia and as described in [33], but with a spatio–temporal component	is derived using the renewal Equation (2)
OpenABM [57]	Stochastic agent–based model calibrated to hospital admissions, ICU bed occupancy, deaths, and vaccinations. Social mixing, test–trace–isolate interventions, different SARS–CoV–2 variants, and progressive vaccination are explicitly modelled	is calculated by directly counting the number of secondary infections that are caused by each primary infection
Covasim [58]	A stochastic agent–based model calibrated to COVID–19 diagnoses, hospital admissions, and deaths related to COVID–19 and modelling progressive vaccine roll–outs. Social distancing and test–trace–isolate interventions are also modelled, and progressive SARS–CoV–2 variants are incorporated	is calculated by directly counting the number of secondary infections that are caused by each primary infection
